# Pharmacokinetic profiles of sertraline in pregnancy as a predictor of postpartum depressive symptoms

**DOI:** 10.1002/bcp.70283

**Published:** 2025-10-07

**Authors:** Sílvia M. Illamola, Zachary N. Stowe, Marc L. Kalin, Michael D. Evans, Catherine M. Sherwin, Maged M. Costantine, D. Jeffrey Newport, James C. Ritchie, Angela K. Birnbaum

**Affiliations:** ^1^ Experimental and Clinical Pharmacology Department University of Minnesota Minneapolis MN USA; ^2^ Department of Psychiatry University of Wisconsin at Madison Madison WI USA; ^3^ Clinical and Translational Science Institute University of Minnesota Minneapolis MN USA; ^4^ Internal Medicine, UWA Medical School The University of Western Australia Perth Western Australia Australia; ^5^ Differentia Bio, South San Francisco San Francisco CA USA; ^6^ Division of Maternal Fetal Medicine, Department of Obstetrics & Gynecology The Ohio State University Wexner Medical Center Columbus OH USA; ^7^ Departments of Psychiatry & Behavioral Sciences and Womens Health The University of Texas at Austin Dell Medical School Austin TX USA; ^8^ Emory University School of Medicine Atlanta GA USA

**Keywords:** antidepressants, pharmacokinetics, pregnancy, sertraline

## Abstract

**Aim:**

To characterize pharmacokinetic changes of sertraline and its metabolite during pregnancy and postpartum, and their relationship to maternal postpartum depressive symptoms.

**Methods:**

This was a prospective observational, longitudinal study of pregnant women with a major depressive disorder treated with sertraline (N = 185 women, 205 pregnancies). Women were enrolled at <16 weeks' gestation and followed at 4‐8 week intervals throughout pregnancy and the first postpartum year. Baseline measures included structured clinical interviews and demographic information. Drug and metabolite concentrations and psychometric measures (study outcomes) (ie, Hamilton Rating Scale for depression – 17 item, Beck Depression Inventory, Edinburgh Postnatal Depression Scale [EPDS], Clinical Global Impression [CGI]) were measured at follow‐up visits. Serum sertraline and N‐desmethylsertraline exposure were reported asstandardized 24‐h concentration‐to‐dose (C/D) and parent to metabolite (P/M) ratios. Linear mixed‐effects and latent trajectory models were used to characterize longitudinal patterns in concentration measures across pregnancy and postpartum, and their association with study outcomes.

**Results:**

Mean 24‐h C/D ratios showed high variability throughout pregnancy and postpartum that were characterized by three trajectories for sertraline and five for N‐desmethylsertraline and P/M ratio corresponding to different sertraline pharmacokinetic profiles. At postpartum, sertraline drug exposure was inversely associated with higher EPDS score (*P* < .05), while N‐desmethylsertraline exposure was associated with higher scores for all measured depression scales (*P* < .001). Higher P/M ratios had higher CGI scores (*P* < .05) postpartum.

**Conclusion:**

Sertraline pharmacokinetic profiles varied across pregnant women and were associated with postpartum depressive symptoms. The use of therapeutic monitoring may provide clinical insight that can be useful for identifying patients with a potential toward depressive symptoms.

**What is already known about this subject**

- Pregnancy‐induced physiological changes alter medication pharmacokinetics, with some pharmacokinetic changes persisting in the postpartum period. Sertraline is a commonly prescribed selective serotonin reuptake inhibitor during pregnancy and postpartum, with limited data characterizing the relationship between pharmacokinetic changes during pregnancy and maternal postpartum or perinatal outcomes.

**What this study adds**

- We identified distinct individual groups defined by their pharmacokinetic trajectory across pregnancy and the postpartum period. This study included outcome measures and the results indicate that individuals exhibit different postpartum depression outcomes based on their pharmacokinetic trajectories.

## 1 INTRODUCTION

Prescribed medications during pregnancy have been reported to range from 27% to 93%^1^ and have increased over recent decades in the United States.^1^ For antidepressants the use changes geographically, ranging from 1.3% (Australia) to 5.5% (United States), with an international prevalence estimate of 3.3% (65% confidence interval [CI] 2.3‐3.7).^2^ Selective serotonin uptake inhibitors (SSRIs) are among the top three most commonly prescribed medications in the general population.^3^

Depression during pregnancy affects up to 20% of women^4^, ^5^ and, if left untreated, there is a risk of adverse outcomes for both mother (eg, more risk of developing postpartum depression)^6^ and child (eg, prematurity, low birth weight), with some effects on the child that can persist into adolescence (eg, social development).^7^, ^8^, ^9^ Changes in physiology during pregnancy can result in significant changes in drug exposure that can lead to unpredictable changes in outcomes,^10^, ^11^, ^12^ therefore pregnant individuals and their foetuses are still at risk of adverse outcomes, making treatment management challenging. Understanding pharmacokinetic (PK) changes during pregnancy is critical in guiding maternal dosing decisions. Additionally, quantification of between‐subject variability in drug exposure that accounts for variation in medication concentrations across pregnancy and postpartum may be a determinant of the maternal daily dose requirements to prevent disease‐related symptoms and improve maternal and child health. Therapeutic drug monitoring can be a helpful tool for monitoring drug concentrations and adjusting doses during pregnancy, and therefore it can be instrumental in optimizing maternal antidepressant outcomes rather than solely rely on clinical judgement.

Sertraline is the most frequently prescribed SSRI antidepressant in pregnant women.^1^ Sertraline has an elimination half‐life (*t*_1/2_) ranging from 22 to 36 h in adults. It is extensively metabolized to N‐desmethylsertraline, which contributes 5‐10% of the pharmacological effect by cytochrome P450 (CYP).^13^, ^14^ Sertraline metabolism is primarily driven by CYP3A4, CYP2C9 and CYP2C19 at high in vitro sertraline concentrations, while CYP2D6 and CYP2B6 are the predominant enzymes at lower concentrations.^14^, ^15^, ^16^ Sertraline and N‐desmethylsertraline cross the human placenta to some extent, allowing for foetal exposure (eg, umbilical cord concentrations are highly correlated with maternal serum concentrations).^17^, ^18^, ^19^ There are discordant data on changes in sertraline concentrations across pregnancy.^20^, ^21^, ^22^, ^23^, ^24^ Notably, in a previous study, only individuals with poor and intermediate CYP2C19 activity showed a decrease in the concentration‐to‐dose ratio (C/D) for sertraline during pregnancy, and the authors proposed this could lead to subtherapeutic sertraline concentrations in this group of individuals.^24^ Previous studies are limited by small sample size and lack of outcome data.

Despite the high frequency of depression during pregnancy, potential adverse outcomes and the relatively common use of antidepressants during pregnancy, there are sparse data on optimal dose management strategies during pregnancy and the postpartum period, potentially limiting the ability to maximize maternal therapeutic response while minimizing maternal and neonatal adverse effects. Some alterations in sertraline pharmacokinetics during pregnancy persisting in the postpartum period may affect maternal, obstetrical and neonatal outcomes, and have not been systematically investigated. The objective of this study was to characterize the PKs of sertraline and N‐desmethylsertraline across pregnancy and examine the potential relationship to the severity of postpartum depression outcomes in a large population of pregnant women.

## 2 METHODS

### 2.1 Study design

This observational, prospective, longitudinal study was performed at the Emory Women's Mental Health Program at Emory University School of Medicine from January 2002 to August 2012 (recruitment and drug analyses). Women (aged 18‐46 years) with a history of neuropsychiatric illness who were planning pregnancy or already pregnant were considered for inclusion. All participants were enrolled before 16 weeks' gestation based on last menstrual period (LMP). Inclusion criteria included (1) fulfilling Diagnostic and Statistical Manual of Mental Illness – IV criteria for Major Depression Disorder; (2) treated with sertraline, taken once daily; and (3) a singleton pregnancy. Women were excluded for (1) active suicidal and/or homicidal ideations; (2) psychotic symptoms; (3) a primary diagnosis of schizophrenia; (4) currently active eating disorders; (5) active substance use disorder within 6 months of LMP; (6) a positive urine drug screen; (7) an illness requiring treatment that can influence outcomes (eg, asthma, autoimmune disorders); and (8) an abnormal thyroid‐stimulating hormone or clinically significant anaemia. We additionally excluded (1) women enrolled during the pre‐pregnancy period who were planning pregnancy but did not conceive and (2) serum concentrations collected at delivery, as the timing of the administration of medications and blood draw during delivery was highly inconsistent.

At enrolment, all subjects completed a structured clinical interview for diagnosis (SCID),^25^ a medical, obstetrical and psychiatric history, and a history of early trauma and life events, as well as social‐economic information. Participants were followed at 4‐8‐week intervals through 12 months postpartum. At each follow‐up visit, participants completed self (ie, Edinburgh Postnatal Depression Scale [EPDS], Beck Depression Inventory [BDI]) and clinician‐rated (ie, Hamilton Rating Scale for depression – 17 item [HRSD‐17], Clinical Global Impression [CGI]) scales for depressive symptoms, information on all prescription and over‐the‐counter medications (ie, daily dose, frequency, time taken), and maternal blood draw collection. Because follow‐up visits were not scheduled at uniform times across individuals (ie, at 4‐8 week intervals), the timing and frequency of concentration sampling and outcome assessments varied between individuals. No treatment decisions (ie, medication selection, dose) were made as part of study participation, and subjects could withdraw at any time. The Institutional Review Board Human Subjects Committee at Emory University approved the study. Written informed consent was obtained from all participants before participation in the study.

### 2.2 Pharmacokinetic sampling and bioanalysis

All blood samples were obtained after maternal sertraline concentrations had attained a steady state (>7 days of the treatment on a fixed dose). Maternal blood samples collected at study visits were processed, coded, entered into a secure study‐specific database and stored at −80 °C (−112 °F) until assay. Laboratory personnel were blind to the maternal daily dose and collection time of the samples, and all samples for a given individual were conducted in the same assay.

Briefly, sertraline and N‐desmethylsertraline in serum were extracted using a solid‐phase extraction procedure. The quantification was accomplished by isocratic high‐performance liquid chromatography separation using a Hypersil (C8) column (2 × 100 mm, i.d., 3 μm particle size) (Keystone Scientific), followed by ultraviolet detection (215 nm). The mobile phase consisted of 0.02 M potassium phosphate monobasic, 38% acetonitrile (pH 6.2) at a 0.6 mL/min flow rate. A five‐point standard curve and two quality control specimens were included in each assay with a lower limit of quantitation of 2.0 ng/mL. Average coefficients of variation were 5% intra‐assay and 10% inter‐assay at sertraline and N‐desmethylsertraline concentrations of 75 and 300 ng/mL.

### 2.3 Statistical analysis

The database was reviewed for missing data and possible errors by visual and systematic inspection of variables. Visits missing maternal daily dose and/or time after dose were excluded from the current study analyses. Sertraline adherence was determined by maternal interview and via concentration measurements. Records were excluded if both sertraline and N‐desmethylsertraline concentrations were below the lower limit of quantification (BLOQ) (< 2 ng/mL). Samples BLOQ included in the analysis were set to half the BLOQ for statistical analysis. In addition, samples obtained more than 25 h after taking the daily dose were excluded.

Study participation did not influence clinical decision‐making, and maternal daily sertraline dose adjustments, if any, were determined clinically and varied across individual patients during pregnancy and the postpartum period. The C/D (ng/mL/mg/day) ratio for sertraline and N‐desmethylsertraline was used to allow sertraline exposure comparison across individuals. The ratio of sertraline (parent) to N‐desmethylsertraline (metabolite) (P/M) was calculated to provide an assessment of metabolism. To adjust for time after dose, we first estimated the relationships between time after dose and proportional changes in C/D values by fitting smooth curves using thin plate regression splines of log parent C/D and log metabolite C/D by hours after dose.^26^ Then, the expected proportional C/D change between the observed time after dose and 24 h after dose was applied to the observed values to calculate standardized 24‐h C/D values. To characterize 24‐h C/D across the perinatal period, we grouped gestational age (GA) during pregnancy and early postpartum (ie, until 12 weeks after delivery) by 4‐week intervals (eg, 0‐4 weeks, 4.1‐8 weeks). Pre‐pregnancy and late postpartum periods (after 12 weeks of delivery) were analysed as separate unique groups: before pregnancy and >12 weeks after delivery, for pre‐pregnancy and late postpartum, respectively.

Standardized 24‐h C/D values were compared by GA groups using linear mixed‐effects models with fixed effects for GA and a random effect for pregnancy nested within participants to account for within‐pregnancy and within‐participant correlation. Time after dose and sertraline daily dose were compared by trimester/postpartum period using linear mixed‐effects models with fixed effects for the trimester/postpartum period and a random effect for pregnancy nested within participants. The daily dose was log‐transformed for analysis. Latent trajectory models were fitted to characterize longitudinal patterns in standardized 24‐h C/D values throughout the pregnancy and postpartum observation period. For that purpose, only individuals with at least two drug concentration measures throughout pregnancy and/or postpartum were included in the analysis. A range of models was fitted with the number of latent trajectory classes varying from 1 to 6, the functional form as quadratic or cubic, and the within‐class variance constrained or unconstrained to be equal across classes. Models were compared using the following criteria: integrated completed likelihood, Akaike information criterion, Bayesian information criterion and the distribution of posterior class membership probabilities. The final models selected were quadratic, unconstrained models with three (parent) or five (metabolite, P/M ratio) latent classes.

Postpartum depression scores (eg, EPDS, BDI, HRSD‐17, CGI) were compared between latent trajectory classes using the multiple pseudo‐class draws method to account for uncertainty in trajectory class membership; 50 data sets were constructed with trajectory classes assigned stochastically based on the posterior class membership probabilities for each pregnancy.^27^ Depression scores were compared between classes in each data set using robust linear models with a cluster term for participants to account for participants with multiple pregnancies, and the results were pooled using Rubin's method.^28^ The mean daily dose was compared by trajectory using the same approach. A final confirmatory analysis was also performed using the same inclusion criteria (pregnancies with at least two drug concentration measures) but limited to one (randomly selected) pregnancy per woman. The average standardized 24‐h C/D values for sertraline, N‐desmethylsertraline and the P/M ratio were calculated for each pregnancy. The relationships between average 24‐h C/D values and postpartum depression scores were examined using thin‐plate regression splines in a generalized additive model to allow for nonlinear relationships, and results were reported using *P* values and regression curves with 95% CIs. An additional sensitivity analysis using this approach with C/D values unadjusted for time after dose was also performed. All the statistical analyses were conducted using R version 4.2.2, including the packages mgcv version 1.8‐41, flex mix version 2.3‐18, lme4 version 1.1‐31 and mice version 3.14.0.

## 3 RESULTS

A total of 268 pregnant women, corresponding to 299 pregnancies, were evaluated for inclusion in the current study. From these 299 pregnancies, some of the time points were under one or more of the following criteria and were excluded from the analyses: (1) information regarding time after dose was not available (n = 92); (2) maternal daily dose information was not available (n = 37); (3) both parent and metabolite concentrations were BLOQ (<2 ng/mL) (n = 56); and (4) samples were obtained more than 25 h after the last daily dose (n = 8). Additionally, all records from pregnancy were excluded if the women (a) did not conceive during the study time period (n = 16 pregnancies) or (b) presented a miscarriage (<24 weeks GA) (n = 2 pregnancies). As a result, 215 individuals (239 pregnancies) were considered for analysis. After the exclusion of data collected at delivery, 185 pregnant women (205 pregnancies) remained, with collected data distributed throughout all pregnancy and postpartum periods included in the analysis. From these 185 pregnant women, 167 only had one pregnancy, while 16 individuals had two pregnancies and two individuals had three different pregnancies throughout the study. The majority of women were white (86.5%), non‐Hispanic/non‐Latino (97.3%), with a median age at delivery of 34 years (range 20.6‐46.8 years). The median (range) gestational age at delivery was 38.7 weeks (29‐46 weeks). The median (range) number of serum samples per pregnancy was 4 (1‐16), with 884 serum concentrations for all individuals (Table 1). We compared sertraline daily doses and the time after the last dose administered between the different gestational/postpartum periods. Time after dose was similar across gestational/postpartum periods (*P* = .98), and sertraline daily dose was higher in the third trimester (geometric mean 90.8 mg) and postpartum (geometric mean 97.5 mg) compared to the first trimester of pregnancy (geometric mean 78.0 mg) (*P* < .0001). Median (range) sertraline and N‐desmethylsertraline concentrations over the whole pregnancy were 51.3 ng/mL (1‐895 ng/mL) and 123 ng/mL (1‐2003 ng/mL), respectively. Table 1 shows the median (range) of sertraline and N‐desmethylsertraline concentrations for each pregnancy period. The most frequent medications co‐administered with sertraline were over‐the‐counter medications: vitamins (75.3%), acetaminophen (31.0%), antiacids (18.8%), pseudoephedrine (11.0%) and diphenhydramine (10.2%). The most used prescription‐only medications administered along with sertraline were lorazepam (10.6%), promethazine (9.8%), ondansetron (9.4%) and zolpidem (7.8%).

**TABLE 1:** Dose and drug concentration characteristics by pregnancy period.

	Total	Pre‐pregnancy (−52 to −0 GAw)	1st TRIM (0 to 13/7 GAw)	2nd TRIM (14 to 27/7 GAw)	3rd TRIM (28 GAw to delivery)	PP (delivery to 56 weeks)
Individuals	185	21	77	121	118	124
Pregnancies	205	23	85	134	131	136
Daily dose (mg)^a^	100 (12.5‐300)	100 (25‐200)	100 (12.5‐300)	100 (12.5‐300)	100 (25‐300)	100 (25‐300)
Time after last dose (h)^a^	6.8 (0.2‐25)	8.4 (1.2‐23.1)	7.1 (1.5‐24)	6.5 (0.2‐25)	6.6 (0.4‐24.7)	6.9 (0.8‐24.4)
Sertraline concentrations						
N (BLOQ)	884	34 (0)	126 (0)	252 (1)	202 (0)	270 (0)
Median (range)	51.3 (1‐895)	51.7 (4‐252.7)	54.2 (2‐217)	51.0 (1‐439)	49.0 (2‐895)	60.6 (2‐588)
N‐DMS concentrations						
N (BLOQ)	883	34 (2)	126 (2)	251 (2)	202 (1)	270 (2)
Median (range)	123.0 (1‐2003)	108.5 (1‐832)	116.6 (1‐1011)	132.0 (1‐1883)	128.7 (1‐1062)	147.0 (1‐2003)

Abbreviations: BLOQ, below the limit of quantification; GAw, weeks of gestational age; N‐DMS, N‐desmethylsertraline; PP, postpartum; TRIM, trimester.

^a^ Expressed as median (range).

**FIGURE 1:**
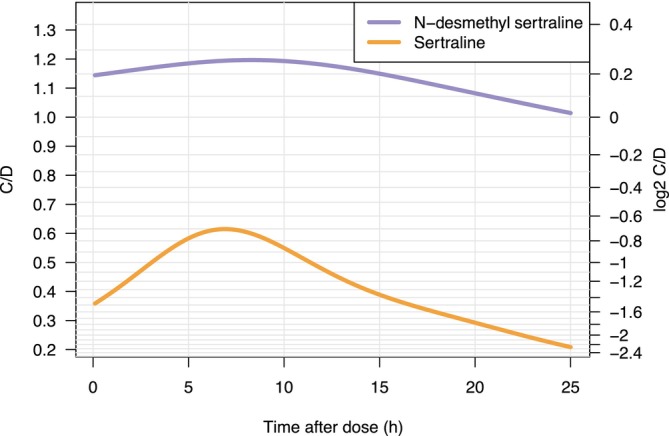
Concentration‐to‐dose ratio (C/D) and log_2_ C/D *vs* time after dose (h) for sertraline (orange) and N‐desmethylsertraline (purple).

**FIGURE 2:**
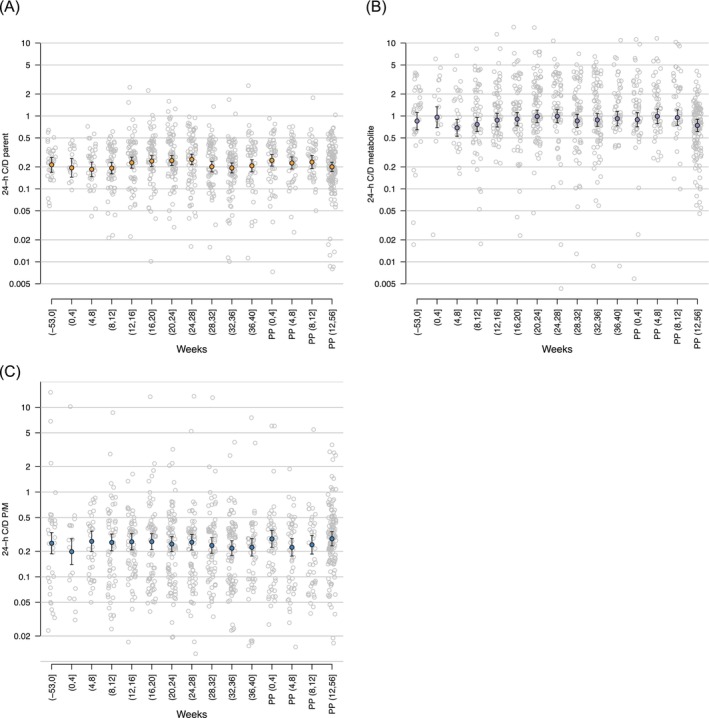
Standardized 24‐h concentration‐to‐dose ratio (C/D) for (A) sertraline (parent), (B) N‐desmethylsertraline (metabolite) and (C) parent to metabolite ratio (P/M) across pre‐pregnancy, pregnancy and postpartum. Data are presented as means with 95% confidence intervals. Numbers in brackets refer to the weeks pre‐pregnancy, pregnancy and after‐delivery (postpartum). PP, postpartum.

The relationship between log_2_ 24‐h C/D and hours after dose, for the parent and the metabolite, showed an increase in 24‐h C/D ratios with a maximum at approximately 7 h after oral dosing, which is in agreement with the maximum serum concentration of sertraline (4‐10 h) (Figure 1).^29^ The mean 24‐h C/D ratios were not significantly different for sertraline (*P* = .14), N‐desmethylsertraline (*P* = .49) or the P/M ratio (*P* = .45) across pregnancy and postpartum. However, the 24‐h C/D standardized ratios for sertraline, N‐desmethylsertraline and the P/M ratio were highly variable across pregnancy and postpartum (Figure 2).

**FIGURE 3:**
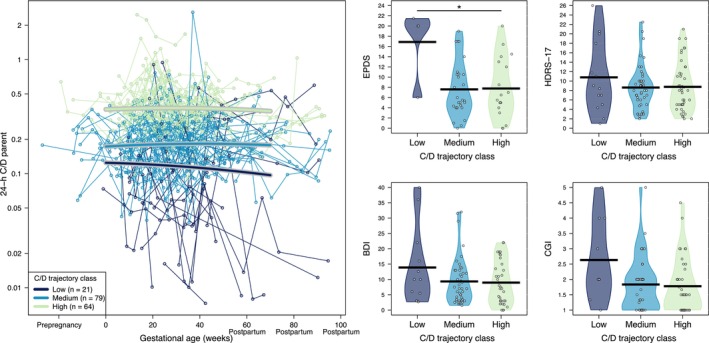
Left: Patterns in standardized 24‐h concentration‐to‐dose ratio (C/D) values throughout the pregnancy and postpartum observation period for sertraline (parent). Right: Individual postpartum depression scales *vs* the three different latent trajectory classes identified for the 24‐h C/D for sertraline (parent). *Statistically significant differences (*P* < .1). BDI, Beck Depression Inventory; CGI, Clinical Global Impression; EPDS, Edinburgh Postnatal Depression Scale; HDRS‐17, Hamilton Rating Scale for Depression – 17 item.

**FIGURE 4:**
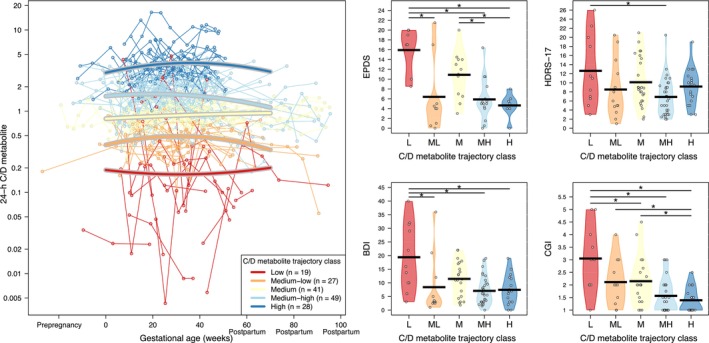
Left: Patterns in standardized 24‐h concentration‐to‐dose ratio (C/D) values over the course of the pregnancy and postpartum observation period for N‐desmethylsertraline (metabolite). Right: Individual postpartum depression scales *vs* the five latent trajectory classes identified for the 24‐h C/D for N‐desmethylsertraline (metabolite). *Statistically significant differences (*P* < .1). BDI, Beck Depression Inventory; CGI, Clinical Global Impression; EPDS, Edinburgh Postnatal Depression Scale; HDRS‐17, Hamilton Rating Scale for Depression – 17 item.

**FIGURE 5:**
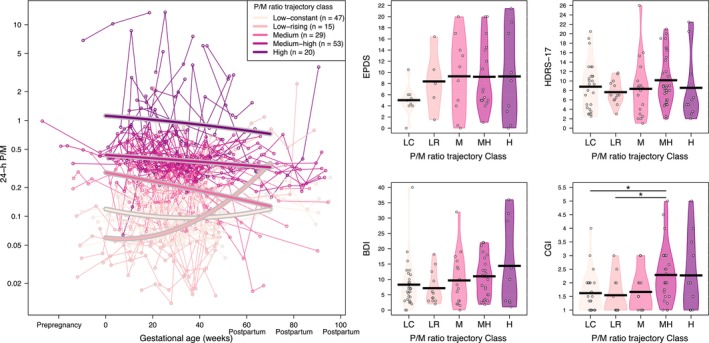
Left: Patterns in standardized 24‐h concentration‐to‐dose ratio (C/D) values over the course of the pregnancy and postpartum observation period for a parent‐to‐metabolite ratio. Right: Individual postpartum depression scales *vs* the five latent trajectory classes identified for the 24‐h C/D for a parent‐to‐metabolite ratio. *Statistically significant differences (*P* < .1). BDI, Beck Depression Inventory; CGI, Clinical Global Impression; EPDS, Edinburgh Postnatal Depression Scale; HDRS‐17, Hamilton Rating Scale for Depression – 17 item; H, high; L, low; LC, low‐constant; LR, low‐rising; M, medium; MH, medium‐high; ML, medium‐low; P/M, parent to metabolite ratio.

Among the 185 individuals, 147 individuals (164 pregnancies) with 843 associated sertraline and N‐desmethylsertraline concentrations had more than one drug concentration available. They were further included in characterizing longitudinal patterns in C/D values throughout the pregnancy and postpartum observation period. From these 164 pregnancies, 94 pregnancies (57.3%) had a change in the daily sertraline dose throughout pregnancy and the postpartum period. Longitudinal patterns in C/D values for sertraline, N‐desmethylsertraline and the P/M ratio were each characterized using latent trajectory models. We identified three different C/D trajectory classes for sertraline (low, medium and high), five for N‐desmethylsertraline (low, medium‐low, medium, medium‐high and high) and five for the P/M ratio (low‐constant, low‐rising, medium, medium‐high and high) throughout the pregnancy and postpartum periods (Figures 3, 4–5). Median values (interquartile range [IQR]) for the parent, metabolite and P/M ratio for each trajectory classes are summarized in Supporting Information Table 1. The mean daily dose by trajectory was statistically different between the low and the other two trajectories for sertraline (Figure S1).

Mean (95% CI) and range values for all psychometric measures for each period are presented in Table 2. Psychometric measures were not available for every individual at all visits. Postpartum depression was assessed using the scales obtained in the early postpartum period (ie, until 12 weeks after delivery) and compared between latent trajectory classes for the C/D ratios for sertraline, N‐desmethylsertraline and the P/M ratios. Generally, the low trajectory groups, those with lower C/D ratios for sertraline and N‐desmethylsertraline, had higher depression scores compared to other trajectories (Figures 3 and 4). However, the lower P/M ratio trajectory group presented lower psychometric measure scores (Figure 5).

**TABLE 2:** Depression scores by period time.

	EPDS		BDI		HDRS‐17		CGI	
	Mean (95% CI)	Range	Mean (95% CI)	Range	Mean (95% CI)	Range	Mean (95% CI)	Range
Pre‐pregnancy	8.6 (6.0‐11.2)	0‐13	10.6 (8.2‐13.0)	0‐30	9.5 (7.7‐11.3)	0‐23	2.1 (1.8‐2.5)	1‐3
First trimester	7.7 (6.3‐9.2)	0‐19	11.5 (10.0‐13.0)	0‐33	10.9 (9.8‐11.9)	0‐27	2.2 (1.9‐2.4)	1‐5
Second trimester	7.7 (6.4‐8.9)	0‐28	10.4 (9.1‐11.7)	0‐55	10.9 (9.9‐11.8)	0‐27	2.1 (1.9‐2.2)	1‐6
Third trimester	7.2 (5.9‐8.6)	0‐26	10.5 (9.1‐11.9)	0‐47	10.8 (9.9‐11.8)	0‐27	1.8 (1.6‐1.9)	1‐5
Postpartum	7.9 (6.8‐9.0)	0‐30	10.3 (9.0‐11.6)	0‐53	9.5 (8.6‐10.4)	0‐27	1.9 (1.8‐2.1)	1‐5

Abbreviations: BDI, Beck Depression Inventory; CGI, Clinical Global Impression; CI, confidence interval; EPDS, Edinburgh Postnatal Depression Scale; HDRS‐17, Hamilton Rating Scale for Depression – 17 item.

Sensitivity analyses using mean C/D values for each pregnancy confirmed the results of the latent trajectory analysis; lower sertraline C/D, lower N‐desmethylsertraline C/D and a higher P/M ratio were associated with higher postpartum depression scores (Supporting Information Figure S2). Similar results were observed when 24‐h C/D values were replaced by C/D values unadjusted for time after dose (Supporting Information Figure S3).

## 4 DISCUSSION

This study included an extensive dataset of sertraline and N‐desmethylsertraline exposure during pregnancy and postpartum along with maternal psychometric depression outcome measures throughout pregnancy and postpartum. We found different sertraline pharmacokinetic profiles across individuals during pregnancy that correlate with postpartum depressive symptoms.

Previous studies examining the pharmacokinetics of sertraline during pregnancy^19^, ^20^, ^21^, ^22^, ^23^ are limited by small sample sizes,^23^ inconsistent metabolite data and limited maternal outcome data. The current study provides the most extensive data set, allowing PK analyses of sertraline in a pregnant population. The results extend previous studies by including outcome measures to further examine potential individual differences and identify groups defined by PK trajectory across pregnancy and the postpartum period.

Although physiological changes occurring during pregnancy can lead to significant changes in drug exposure (pharmacokinetics), this study did not find a significant change in the C/D ratio for both sertraline and N‐desmethylsertraline throughout pregnancy and the postpartum period across the population. The absence of a significance change on C/D ratio over the course of pregnancy and postpartum precluded any definitive guideline suggestions regarding adjustment of maternal daily dose based on the impact of pregnancy. However, scrutinizing data to describe the longitudinal changes that align with individual trajectory groups and potential association with measures of maternal depression raises essential questions regarding therapeutic drug monitoring in pregnancy.

Significant differences between depression postpartum measures and sertraline and metabolite pharmacokinetic trajectories indicate that individuals have different outcomes based on their pharmacokinetic profiles. The evidence of other clinical outcomes associated with particular sertraline pharmacokinetic profiles provides the opportunity for prospective individual drug dose optimization. The use of therapeutic drug monitoring can be useful for identifying patients with more of a potential toward depressive symptoms from the beginning of treatment, thus maximizing efficacy while minimizing side effects. This study supports a more typical therapeutic drug monitoring approach as performed in other clinical specialties, such as neurology (ie, antiseizure drugs), including the C/D ratio for both parent and metabolite. The inclusion of drug concentration monitoring provides an objective measure that can be used to optimize and enhance the importance of different PK trajectories in optimizing maternal treatment. Notably, both parent and metabolite trajectories were associated with depression scores. N‐desmethylsertraline does convey a portion of the purported therapeutic action of sertraline, but this is typically considered a minimal contribution. The consistency of the relationship between parent and metabolite with clinical outcome measures across different trajectory groups would suggest that individual differences in metabolism are related to maternal depressive symptoms in the postpartum period. This important finding has potential direct clinical significance because identifying the pharmacokinetic profile early in treatment may help to individualize sertraline dosing to optimize maternal outcomes. However, the interpretation of an association of a lower P/M ratio with lower depression scores is challenging, given the previously noted correlations. One potential explanation could be the relative contribution of polymorphic enzymes, as genetic variability in CYP enzymes may lead to differences in drug metabolism. Further investigation of P/M ratios and clinical measures is warranted.

The current study has several strengths. To our knowledge, this is the largest sample size of pharmacokinetic studies in pregnancy for antidepressants. Second, the cohort of women is exceptionally well characterized from a diagnostic perspective. Next, the biological samples were collected at several points across pregnancy and the postpartum period. Finally, each biological sample is paired with psychometric data.

There are several limitations and potential confounding factors in our study. Sertraline is metabolized by CYP2C19. However, in our study, DNA information on CYP2C19 was not available and therefore it could not be included in our analysis. Alternatively, although the role of CYP2C19 in managing sertraline and improving maternal outcomes is unknown, the ratio of the metabolite to the parent drug was used and allowed an estimation of the contribution of metabolism. Other weaknesses of the study are the homogeneous cohort that may limit generalization and the ability to assess other factors that could potentially influence pharmacokinetic profiles. Medication adherence and dose timing depended on maternal reports, factors that can influence observed sertraline concentrations. Sertraline is also 98% bound to plasma proteins, and the decrease in plasma proteins during pregnancy could lead to increased unbound sertraline concentrations and further define exposure. Although unbound concentrations could be a significant factor to consider, unbound concentrations of sertraline were not available. It should be noted that previous studies exploring the pharmacokinetics of the unbound fraction of other highly bound drugs (eg, lopinavir, raltegravir) during pregnancy found that unbound concentrations were not significantly changed.^30^, ^31^ Comedications present in this study are unlikely to significantly affect sertraline metabolism either because they are not metabolized by the CYP450 enzyme system (eg, vitamins, lorazepam) or because they are weak inhibitors (eg, diphenhydramine) or inducers (eg, zolpidem) of CYP450 enzymes. Finally, the C/D ratio is helpful to standardize concentrations when administered doses are different to assess overall drug exposure in individuals. This method does not account for time after dose but provides an overall post hoc exposure value per individual that allows exploration of sources of pharmacokinetic variability.

Subsequent analyses will include a population pharmacokinetic analysis that can address most of the confounding factors and limitations of this study. Population pharmacokinetic analysis quantifies variability between (inter‐) and within (intra‐) individuals, as well as interoccasion variability, that can be present between different trimesters of pregnancy. Population pharmacokinetic analysis is especially suitable for sparse sampling studies, which is common in studies that include pregnant individuals.

Our results indicate that sertraline pharmacokinetic profiles vary across pregnant women and that there is a relationship between changes in sertraline exposure (ie, pharmacokinetics) and maternal symptoms (pharmacodynamics) at postpartum with objective clinical measures, providing evidence for the establishment of clinical guidelines relevant to pregnant women.

## AUTHOR CONTRIBUTIONS

*Contributed to writing and editing the manuscript*: Sílvia M. Illamola, Zachary N. Stowe, Marc L. Kalin, Michael D. Evans, Catherine M. Sherwin, Maged M. Costantine, D. Jeffrey Newport, James C. Ritchie, Angela K. Birnbaum. *Research design*: Sílvia M. Illamola, Zachary N. Stowe, Marc L. Kalin, Michael D. Evans, Angela K. Birnbaum. *Performed data analysis*: Sílvia M. Illamola, Michael D. Evans, James C. Ritchie. *Data analysis*: Sílvia M. Illamola, Michael D. Evans, Angela K. Birnbaum.

## CONFLICT OF INTEREST STATEMENT

Silvia Illamola reports no financial relationships with commercial interests. Zachary Stowe has received research support from Glaxo SmithKline (GSK), Janssen, Pfizer, Wyeth, the National Institutes of Health (NIH) and The Center for Disease Control. He has served on speakers' bureaus and/or received honoraria from Eli Lilly, GSK, Pfizer and Wyeth. He has served on advisory boards for GSK, BMS and Reunion Neuroscience. Neither he nor family members have ever held equity positions in biomedical or pharmaceutical corporations. Marc Kalin reports no financial relationships with commercial interests. Michael Evans reports no financial relationships with commercial interests. Catherine Sherwin reports receiving grant support paid to her previous institution from the National Institutes of Health unrelated to the work in this manuscript. This work was completed while she was at Wright State University, Ohio. She is currently employed at the University of Western Australia and Differentia Bio (a US‐based CRO). She is just an employee and has no stock or financial interest in the company. She is also an Editorial board member of BJCP. Maged Costantine reported receiving grant support for work not related to this paper from NICHD, NHLBI, PCORI and the Foundation for the NIH, and personal consulting fees not related to this paper from Diamedica and Siemens Healthcare. He is on the clinical advisory board for Comanche for work related to preeclampsia therapeutics. Jeffrey Newport has received research support from Eli Lilly, Glaxo SmithKline (GSK), Janssen, Sage Therapeutics, Takeda Pharmaceuticals, the Texas Health & Human Services Commission and Wyeth. He has served on speakers' bureaus and/or received honoraria from Astra‐Zeneca, Eli Lilly, GSK, Pfizer and Wyeth. He has served on advisory boards for GSK, Janssen and Sage Therapeutics. He has never served as a consultant to any bio‐medical or pharmaceutical corporations. Neither he nor his family members have ever held equity positions in biomedical or pharmaceutical corporations. James Ritchie reports no financial relationships with commercial interests. Angela Birnbaum reports receiving grant support, paid to her institution, from UCB Pharma, Vireo Health, LLC, Randy Shaver Cancer and Research Foundation, and the National Institutes of Health, holding patent US9770407B2 on parenteral carbamazepine formulation, licensed to Lundbeck, and patent EP12150783A on novel parenteral carbamazepine formulations, licensed to Lundbeck.

## Supporting information


**SUPPORTING INFORMATION TABLE S1** Parent, metabolite and parent‐to‐metabolite ratio by pregnancy period.

**SUPPORTING INFORMATION FIGURE S2** Relationships between standardized 24‐h concentration‐to‐dose ratio (C/D) values and postpartum depression scores for (A) sertraline, (B) N‐desmethylsertraline and (C) parent‐to‐metabolite (P/M) ratio, limited to one pregnancy per woman. EPDS, Edinburgh Postnatal Depression Scale; HDRS‐17, Hamilton Rating Scale for Depression – 17 item; BDI, Beck Depression Inventory; CGI, Clinical Global Impression.

**SUPPORTING INFORMATION FIGURE S3** Relationships between mean 24‐h concentration‐to‐dose ratio (C/D) values uncorrected for time after dose (TAD) and postpartum depression scores for (A) sertraline, (B) N‐desmethylsertraline and (C) parent‐to‐metabolite (P/M) ratio. EPDS, Edinburgh Postnatal Depression Scale; HDRS‐17, Hamilton Rating Scale for Depression – 17 item; BDI, Beck Depression Inventory; CGI, Clinical Global Impression.

SUPPORTING INFORMATION FIGURE S1

## Data Availability

The data underlying this study are not publicly available due to restrictions outlined in the original consent forms, which did not include provisions for data sharing. Additionally, data sharing policies have evolved since the study's approval, and retrospective re‐consent was not feasible. No new data were collected specifically for this study; all analyses were performed on pre‐existing datasets collected during the study's original approval period.
