# Supplementary material for: Pharmacokinetic profiles of sertraline in pregnancy as a predictor of postpartum depressive symptoms
Source: Br J Clin Pharmacol. 2025 Oct 7;92(3):830–9. doi: 10.1002/bcp.70283 (PMC12930024; doi:10.1002/bcp.70283)
Supplement: Supplementary file 1 — SUPPORTING INFORMATION TABLE S1 Parent, metabolite and parent‐to‐metabolite ratio by pregnancy period. [file BCP-92-830-s003.docx]

**Supplemental Table 1.** Parent, metabolite, and parent-to-metabolite ratio by pregnancy period.

| **Measure** | **Observations** | **Pregnancies** | **Trajectory** | **Median [IQR]** |
| --- | --- | --- | --- | --- |
| **24hr C/D parent** | 112 | 21 | Low | 0.10 [0.06, 0.22] |
|  | 383 | 79 | Medium | 0.18 [0.15, 0.24] |
|  | 348 | 64 | High | 0.38 [0.32, 0.44] |
| **24hr C/D metabolite** | 70 | 19 | Low | 0.21 [0.11, 0.37] |
|  | 118 | 27 | Medium-Low | 0.41 [0.35, 0.57] |
|  | 230 | 41 | Medium | 0.85 [0.77, 1.02] |
|  | 285 | 49 | Medium-High | 1.39 [1.22, 1.97] |
|  | 139 | 28 | High | 3.80 [3.16, 4.22] |
| **24hr P/M** | 220 | 47 | Low-Constant | 0.10 [0.08, 0.13] |
|  | 114 | 15 | Low-Rising | 0.07 [0.05, 0.10] |
|  | 166 | 29 | Medium | 0.23 [0.17, 0.26] |
|  | 258 | 53 | Medium-High | 0.36 [0.32, 0.44] |
|  | 84 | 20 | High | 0.73 [0.63, 1.01] |

C/D: standardized 24-hour concentration to dose ratio (C/D); IQR: Interquartile range
