# Supplementary figures and images for: Pharmacokinetic profiles of sertraline in pregnancy as a predictor of postpartum depressive symptoms
Source: Br J Clin Pharmacol. 2025 Oct 7;92(3):830–9. doi: 10.1002/bcp.70283 (PMC12930024; doi:10.1002/bcp.70283)

**A.**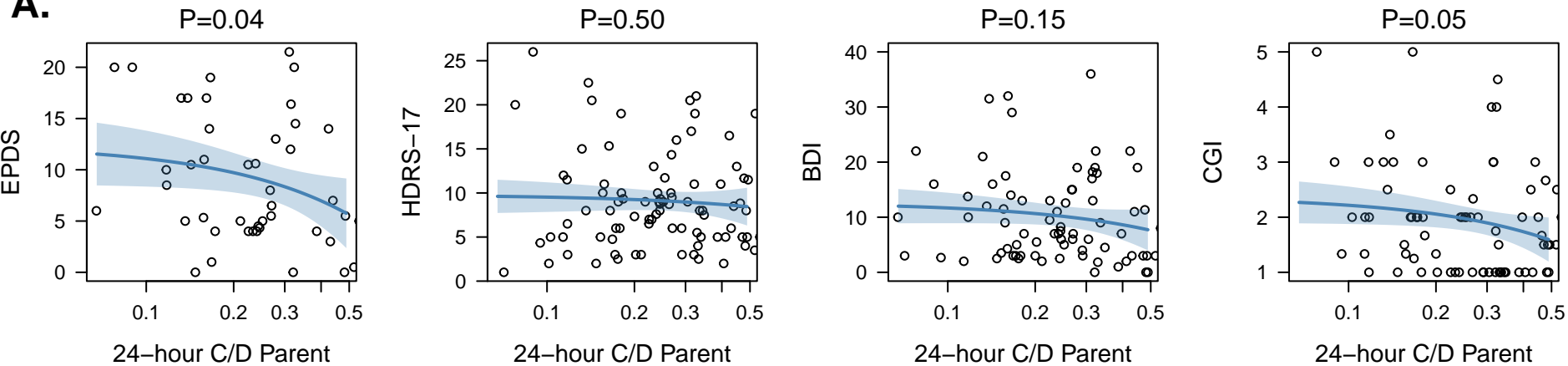**B.**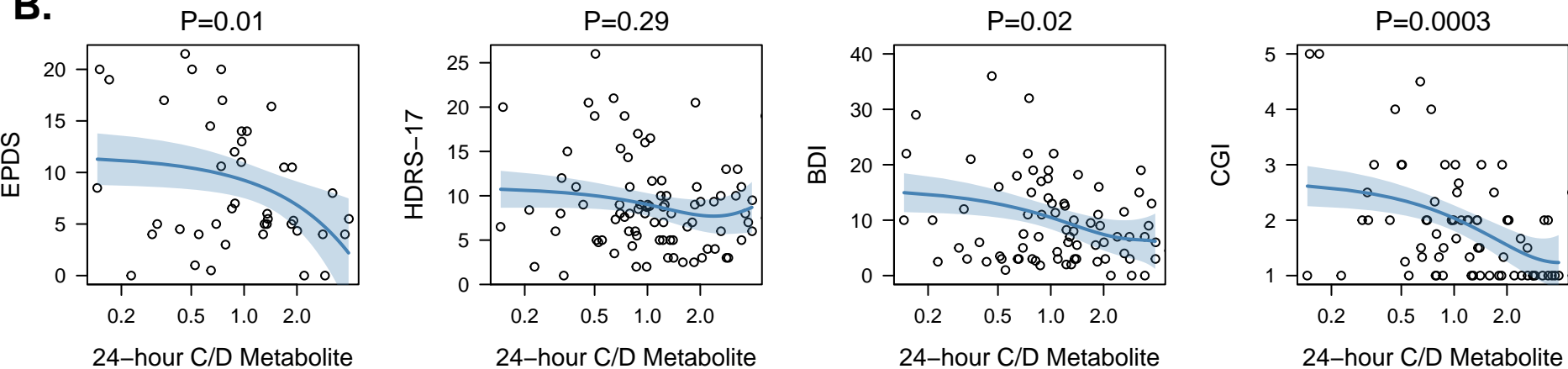**C.**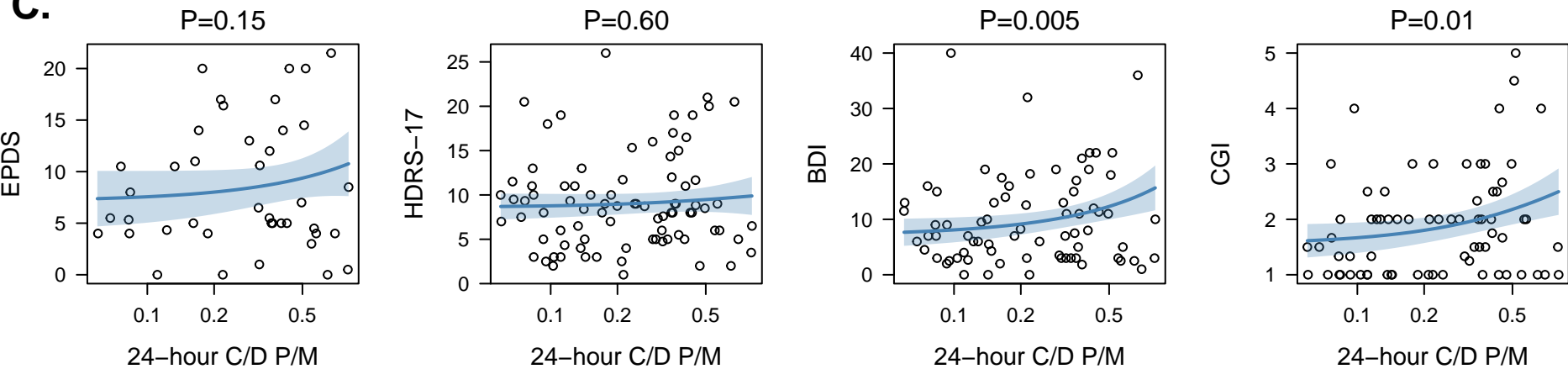

Supplement: Supplementary file 2 — SUPPORTING INFORMATION FIGURE S2 Relationships between standardized 24‐h concentration‐to‐dose ratio (C/D) values and postpartum depression scores for (A) sertraline, (B) N‐desmethylsertraline and (C) parent‐to‐metabolite (P/M) ratio, limited to one pregnancy per woman. EPDS, Edinburgh Postnatal Depression Scale; HDRS‐17, Hamilton Rating Scale for Depression – 17 item; BDI, Beck Depression Inventory; CGI, Clinical Global Impression. [file BCP-92-830-s004.pdf]

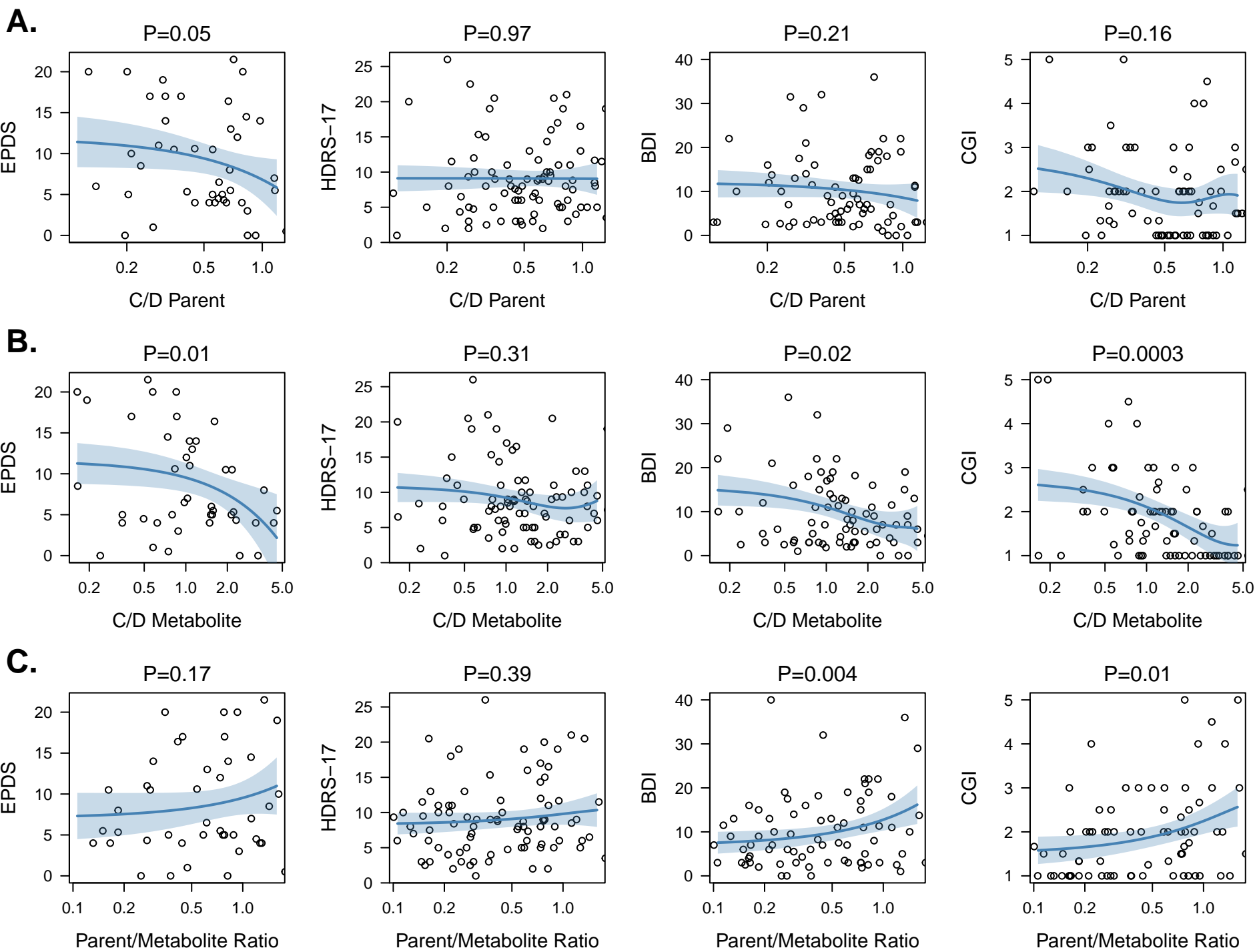

Supplement: Supplementary file 3 — SUPPORTING INFORMATION FIGURE S3 Relationships between mean 24‐h concentration‐to‐dose ratio (C/D) values uncorrected for time after dose (TAD) and postpartum depression scores for (A) sertraline, (B) N‐desmethylsertraline and (C) parent‐to‐metabolite (P/M) ratio. EPDS, Edinburgh Postnatal Depression Scale; HDRS‐17, Hamilton Rating Scale for Depression – 17 item; BDI, Beck Depression Inventory; CGI, Clinical Global Impression. [file BCP-92-830-s002.pdf]

A.

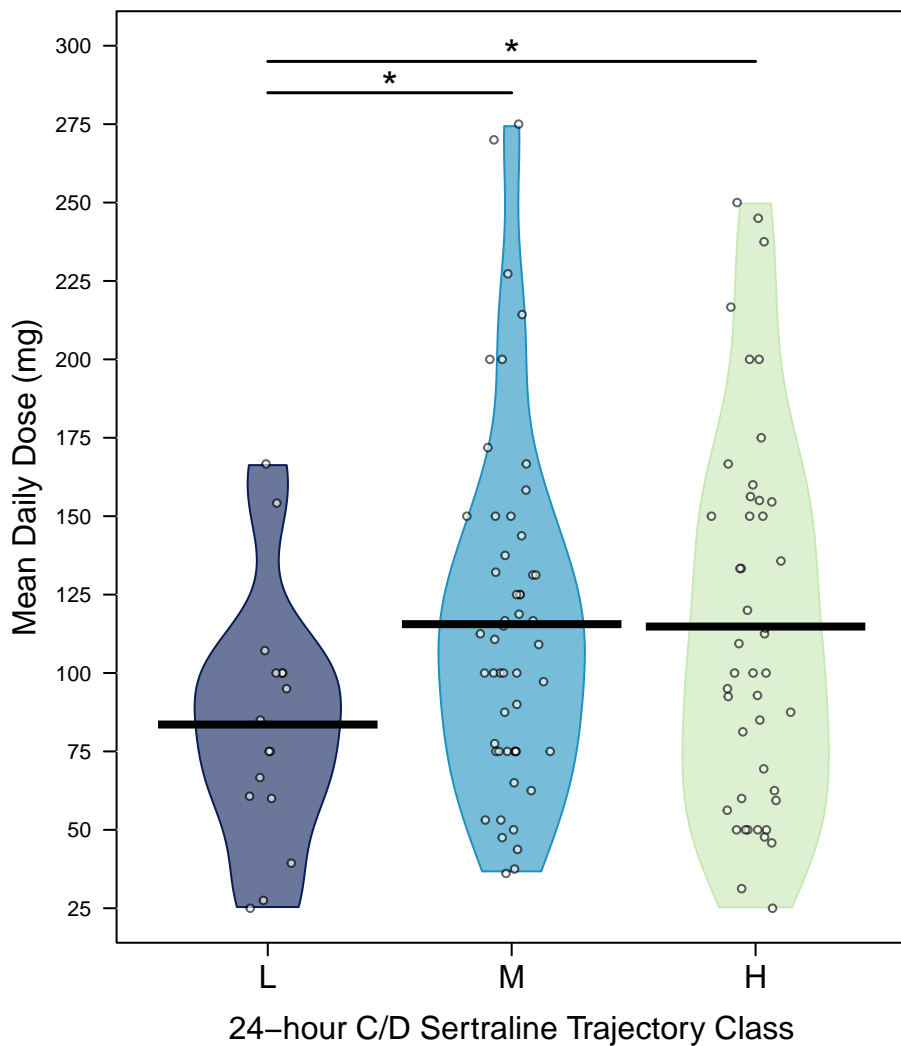

B.

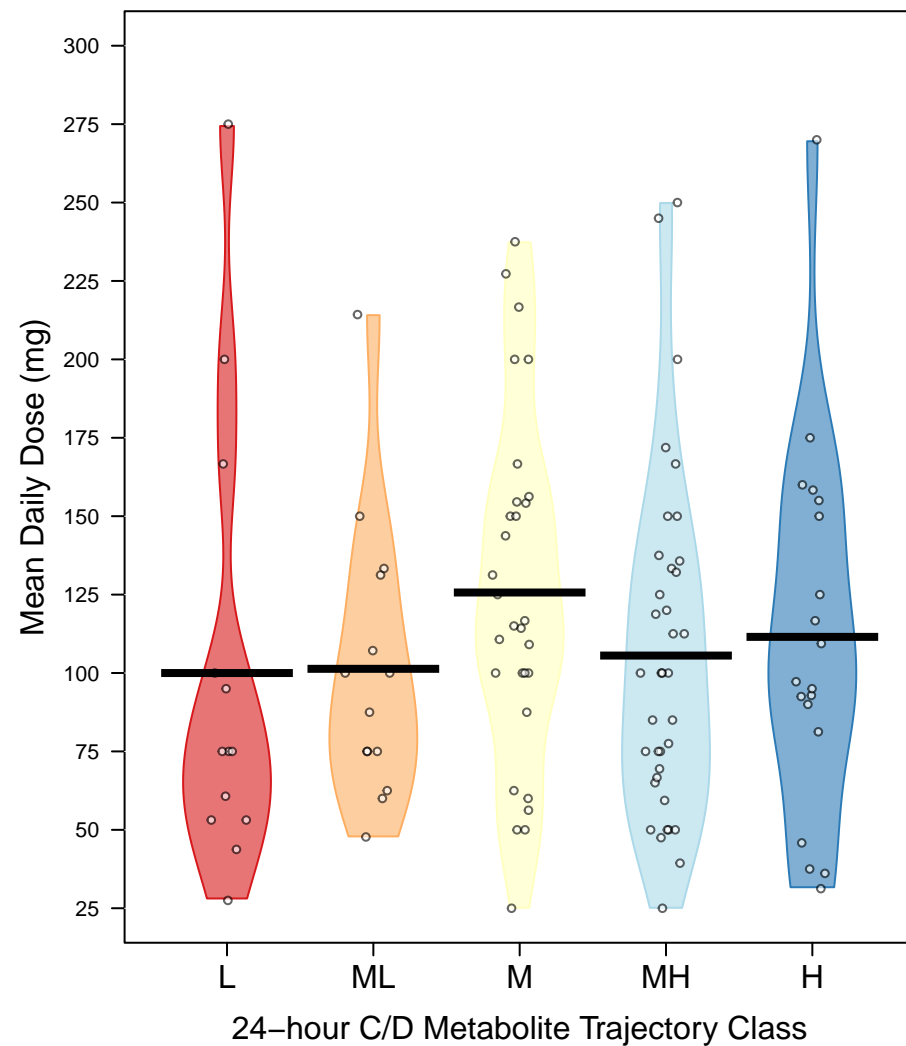

C.

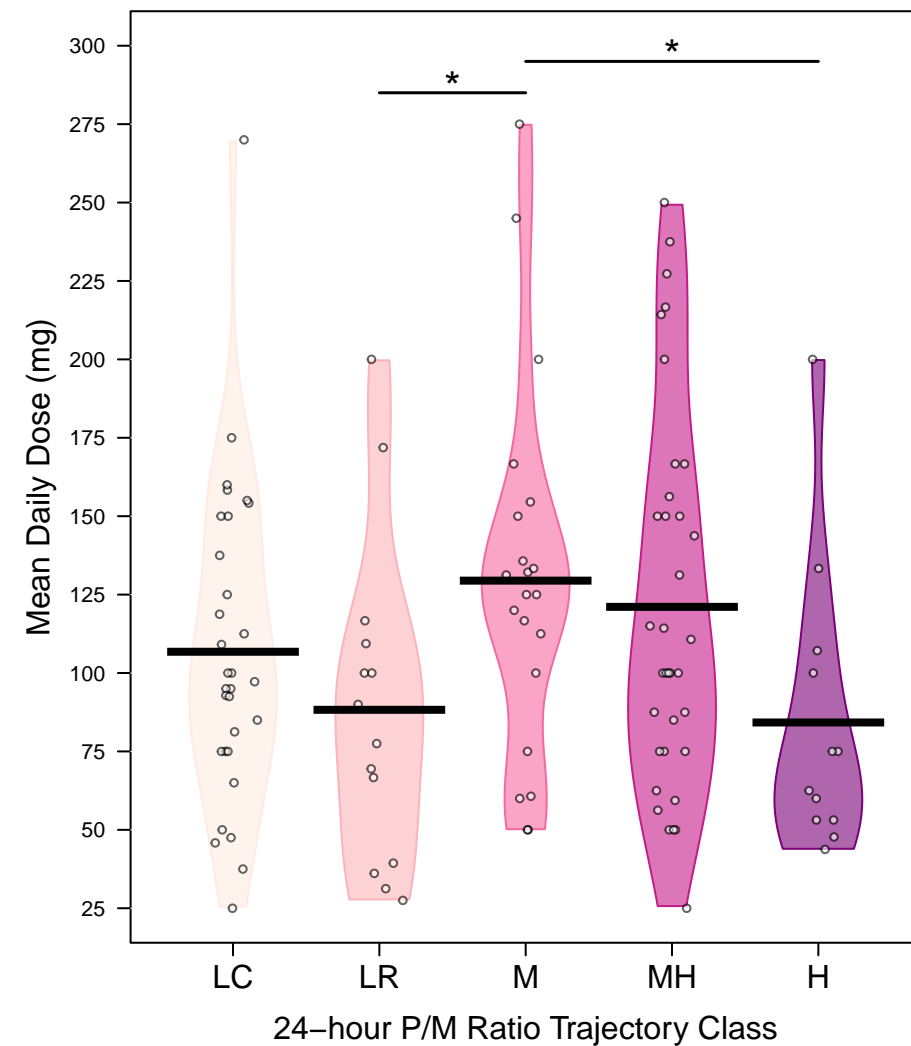

Supplement: Supplementary file 4 — SUPPORTING INFORMATION FIGURE S1 [file BCP-92-830-s001.pdf]
